# Do interest groups bias MPs’ perception of party voters’ preferences?

**DOI:** 10.1177/1354068821997079

**Published:** 2021-04-15

**Authors:** Steven Eichenberger, Frédéric Varone, Luzia Helfer

**Affiliations:** 27212 Université de Genève, Switzerland

**Keywords:** business groups, citizen groups, democracy, interest groups, parliament, party, representation

## Abstract

This study analyses how information provided by different types of interest groups influences the ability of members of parliament (MPs) to accurately perceive the preferences of those citizens who voted them into office. To study how information provision by interest groups affects MPs’ perceptions, we combine unique data from a citizen survey and face-to-face meetings with 151 federal MPs in Switzerland, thus enabling a comparison of actual voter preferences with MPs’ estimations of these preferences. Ties to citizen groups, as self-reported by MPs in our survey, relate to more accurate perceptions by MPs, even when controlling for MPs’ partisan affiliation. Ties to business groups, as declared in the official registry, relate to less accurate perceptions. These findings suggest that interest groups can both tighten and weaken MPs’ link to their party voters, which might have repercussions on substantive representation and democratic accountability.

## Introduction

Interest groups represent a most pervasive fact of modern democracies. They are a vital part of representative politics as key intermediary actors between voters and members of parliament (MPs). Interest groups aggregate the policy preferences of (segments of) MPs’ constituencies and they try to influence the decision-making process by delivering targeted information to representatives. Empirical evidence shows that MPs consider interest groups as an important information source, next to direct contacts with citizens and the traditional media (Wonka, 2017).

The literature describes the linkages between individual MPs and interest groups as exchange relationships (Berkhout, 2013). MPs grant interest groups access to the parliamentary decision-making venue (e.g. legislative committees), or even commit themselves to support some policy proposals advocated by the groups’ lobbyists. In exchange, interest groups provide like-minded MPs with information about group members’ preferences, campaign contributions, electoral support, policy expertise, and political intelligence about the law-making process. Providing such information to like-minded allies is important and highly valued as a ‘legislative subsidy’ by resource-constrained MPs (Hall and Deardorff, 2006: 75).

Studies show that these exchange relationships between interest groups and individual MPs have far reaching consequences. They have an impact on MPs’ agenda-setting activities (Fischer et al., 2019), parliamentary oversight of government (Varone et al., 2018), and eventually also on MPs’ policy decisions and thus on substantive representation (Giger and Klüver, 2016; Gilens and Page, 2014; Klüver and Pickup, 2019). Importantly, these latter studies show variation across interest groups. Business groups, which defend sectional interests, appear to weaken the link between MPs and their voters. Citizen groups, on the other hand, which defend broadly shared and ‘diffuse’ interests and which seek to establish public rather than club goods, strengthen this link.

Thus, as MPs rely on the information provided by interest groups, they must pay heed to the concerns of both their electorate at large and the interest groups supporting them. Rather than focusing on the factors that might lead MPs to favour one master over the other, this article questions whether collaboration with interest groups actually affects MPs’ ability to distinguish between the preferences of their electorate, on the one hand, and the interest groups supporting them, on the other. Does reliance on information provided by interest groups make MPs lose sight of their party voters’ opinions, that is, do they decrease MPs’ perceptual accuracy? Does it make a difference whether MPs rely on information provided by citizen rather than business groups?

Extant research on how interest groups inform MPs’ perceptual accuracy has been scarce and very much focused on the US context, and thus on a majoritarian electoral system and district/state level public opinion (Broockman and Skovron, 2018; Hedlund and Friesema, 1972; Hertel-Fernandez et al., 2019). These show that exposure to business groups, which clearly defend sectional interests and thus minority positions, decreases legislative staffers’ perceptual accuracy of public opinion within a given electoral district (Hertel-Fernandez et al., 2019). However, and especially in proportional representation electoral systems, MPs (and their staffers) should also keep an eye on the opinion of their party electorate, which voted them into parliament and to whom they are accountable. It is less clear whether links to business groups decrease MPs’ perceptual accuracy when they respond to a party electorate which could generally be more business friendly than the overall electorate.

Building on these existing studies, we thus focus explicitly on actively elected MPs’ perceptual accuracy of the opinion of their party electorate. Our research design compares data from a survey among voters to MPs’ estimation of their voters’ position. We asked MPs to estimate the opinion of their party electorate on a number of very specific policy statements (e.g. ‘Switzerland should only accept well-educated immigrants’; ‘The pension age needs to be raised to 67’; or ‘Switzerland needs to buy new fighter jets’). By comparing actual public opinion with MPs’ estimations, we can assess MPs’ perceptual accuracy for each statement. We then pair this information with data on the ties these MPs entertain with different interest groups. We rely on both self-reported ties and ties declared in the mandatory, official registry, according to article 11 of the Swiss Parliament Act. Integrating these various information sources allows us to gauge whether links to relevant interest groups affect MPs’ perceptions of their voters’ opinions on specific policy statements. We seek to understand how ties to different types of interest groups moderate the effect of MPs’ efforts to inform themselves about their electorate’s preferences.

With this research design, we focus on the very core of political representation: MPs’ perceptions of their party voters’ opinion. Moreover, in contrast to previous studies, which were limited in the number of policy issues they could cover, often because they had to draw on existing data for information on the opinion of citizens from ballots or other surveys, we use nine different issues, for which we formulated two specific policy statements each. According to the self-reported ties (in the survey), citizen groups have a positive impact on MPs’ perceptual accuracy, even when controlling for MPs’ partisan affiliation. When focusing on ties declared in the official registry, we do not find citizen groups to increase MPs’ perceptual accuracy. However, business groups appear to have a negative impact on the extent to which MPs correctly perceive their electorate’s preferences, again when controlling for partisan affiliation.

## Theoretical framework

Having accurate perceptions of what the public, and particularly the electorate, thinks about specific policy statements is key to representation. MPs need to have a good understanding of what their party electorate wants in order to adopt policies that are congruent with voters’ preferences (Lax and Phillips, 2012), to explain and justify why they have a diverging policy position (Esaiasson and Wlezien, 2017; Thomassen and Schmitt, 1999), or to be responsive to opinion changes (Stimson et al., 1995). Together with MPs’ willingness to represent their party electorate, the ability of MPs to correctly perceive what party voters wish is thus a necessary condition for substantive political representation (Pitkin, 1972).

Interest groups are likely to play an important role not just in affecting overall policy outcomes, but also in shaping politicians’ perceptions. In their seminal article on perceptual accuracy, Miller and Stokes (1963: 54) postulated that MPs’ information environment might be biased since engagement in their electoral district ‘inevitably puts them in touch with organized groups and with individuals who are relatively well informed about politics’. Indeed, interest groups are often part of MPs’ electorates, which gives them the right to contact MPs and provide them with both technical expertise and political intelligence. However, only few studies on perceptual accuracy to date have explicitly incorporated interest groups in their research design.

Hedlund and Friesema (1972: 736, 742–743), among the first to study the link between interest groups and MPs’ perceptual accuracy, did not find MPs collaborating with interest groups to be less accurate. However, they did not distinguish between different types of interest groups. More recently, and focusing on candidates for state-level US elections, Broockman and Skovron (2018: 559) emitted the hypothesis that the particularly vocal and organized base of conservative groups (e.g. the members of the National Rifle Association, members of anti-abortion groups) leads Republican politicians to overestimate public support for conservative positions. While such ‘conservative’ groups might also comprise certain business interest associations, Broockman and Skovron thus suggested that conservative citizen groups decrease (particularly Republican) politicians’ perceptual accuracy.^1^

To our knowledge, only one study has tested whether interest groups affect MPs’ perceptual accuracy, albeit with MPs’ staffers and not the representatives themselves. Hertel-Fernandez et al. (2019), focusing on the perceptual accuracy of MPs’ senior staffers in the US, find that reliance on mass-based, citizen groups for policy information, operationalized as labour union density in the staffers’ electoral district, actually increases staffers’ perceptual accuracy. This finding resonates with research on interest groups’ alignment with public opinion, which shows that citizen groups most often defend majoritarian positions (Flöthe and Rasmussen, 2019; Giger and Klüver, 2016). Reliance on business groups, however, operationalized as campaign contributions from business groups, decreases legislative staffers’ (and arguably also MPs’) perceptual accuracy (Hertel-Fernandez et al., 2019).

Overall, research on the link between MPs’ perceptual accuracy and interest groups is surprisingly scarce and mostly focuses on the US context and thus on public opinion at the district or state level. However, from a normative standpoint, and without adhering to an entirely populist view of representative democracy, MPs should be able to accurately assess the opinion of their party electorate in order to make an informed decision in parliament (Thomassen, 1999). Especially in proportional representation electoral systems, it is important for MPs to differentiate between minority positions at the district level and majority positions within their party electorate. District-level and party electorate opinions are not necessarily congruent. Interest groups’ impact on MPs’ perceptual accuracy might thus vary, depending on whether the focus is put on the district-level or party electorate opinion.

Concretely, whether policy information provided by business groups indeed decreases right-wing (pro-business) MPs’ perceptual accuracy remains open to scrutiny. It crucially depends on the extent to which business groups’ policy positions resonate among the wider right-wing party electorate. Certain peak-level business interest associations might indeed defend positions supported by a majority of right-wing voters. However, in highly diversified economies, which might be further characterized by the duality between their export and domestic-market oriented sectors, most business groups are likely to defend minority positions, even among right-wing voters. The positions defended by business groups thus represent biased estimators and are likely to negatively affect an initial assessment established on the basis of less biased information (newspapers, peers, party line, past experience, general opinion polls). This argument also extends to left-wing MPs collaborating with business groups.

Citizen groups, on the other hand, defend positions that are, by definition, broadly shared and often tap into the broad core issues defended by certain parties. When focusing on the district-level opinion, citizen groups appear to increase MPs’ perceptual accuracy (Hertel-Fernandez et al., 2019). When focusing on the party electorate opinion in a proportional representation electoral system, we also expect citizen groups to increase MPs’ perceptual accuracy, regardless of the interest groups’ political leaning. That is, we expect ‘leftist’ citizen groups (e.g. environmental groups, trade unions) to defend positions which strongly resonate among the left-wing party electorate, and in the same vein, we expect ‘rightist’ citizen groups (e.g. anti-immigration or anti-abortion groups) to provide right-wing MPs with a fairly accurate idea of right-wing party voters’ preferences. This does not mean that leftist (rightist) interest groups shun right-wing (left-wing) MPs. On the contrary, De Bruycker (2016) showed that interest groups, be they business or citizen groups, frequently contact MPs holding different views in an attempt to persuade them. And such contacts, which are difficult to capture in the absence of a lobbying registry, might also affect MPs’ perceptual accuracy, but do not present the focus of the current analysis.

Interest groups in fact rarely provide MPs with actual information about their members’ preferences (Chalmers, 2013: 51). Interest group scholars agree that policy expertise represents the crucial ‘access good’. We consider that the provision of such expertise can lead MPs to associate the position defended by the group to the position held by their respective electorates. In sum then, we expect information drawn from citizen groups to increase MPs’ perceptual accuracy as citizen groups’ positions often correspond to the majority position within MPs’ party electorate (H1). In contrast, information drawn from sectional business groups should decrease MPs’ perceptual accuracy as they lead MPs to confound the position of a minority within their electorate with the party electorate as a whole (H2).

## Data and methods

In the following, we first detail the construction of our dependent variable, MPs’ perceptual accuracy of the party electorate opinion on a set of concrete policy statements. Secondly, we explain how we devised our main independent variable, MPs’ ties to interest groups, relying on both validated data drawn from an official registry and self-reported. Thirdly, we introduce four sets of control variables.^2^

### Dependent variable: MPs’ perceptual accuracy at the policy statement level

We draw on two sources of evidence to assess MPs’ perceptual accuracy: (1) survey measures asking MPs to make an estimation of where the party voters stand with regard to policy statements, and (2) data about party voters’ actual preferences with regard to the same statements from a population survey. We used a total of 18 policy statements across nine different issues. In order not to overburden respondents, MPs and citizens were assigned to one of two batches, each comprising nine statements (see Table A1 in the Online Appendix for a full list).

The public opinion survey allowing us to gauge the opinion of the MPs’ party electorate was conducted among the Swiss population in French and German between June and August 2018, excluding the Italian-speaking part of Ticino. Of the otherwise nationally representative probability sample of 10,268 addresses, obtained from the Swiss Federal Statistical Office, a response rate of 46% (N = 4,677) was attained. The respondents are largely representative for age, gender, education and party affiliation. To measure the party electorate opinion, we simply asked respondents to position themselves on a five-point scale with regard to each statement: absolutely disagree, rather disagree, rather agree, fully agree and one option labelled as ‘undecided (neutral or no opinion)’. For each policy statement and party, we then aggregated those absolutely or rather agreeing per party to obtain a score for the percentage of party voters that absolutely or rather agrees with the statement. To identify the party voters, we asked respondents to indicate the party they had voted for in the most recent general elections in 2015. For respondents who did not remember, we complemented this with the party they would vote for if elections were held today. We excluded the smallest parties which only have one to two seats in the national parliament and whose electorate is not sufficiently represented in our data to calculate reliable estimates. We have between 51 and 425 observations per party per statement (M = 307, SD = 114) to gauge the opinion of the party electorate. Detailed information for each party electorate can be found in the appendix (see Table A2 in the Online Appendix).

The MPs survey was conducted among elected representatives in both chambers of the federal Parliament (i.e. National Council and Council of States, excluding representatives from Ticino). The surveys were completed by politicians on a tablet during face-to-face meetings between August and October 2018. Overall, 151 of the 236 politicians participated in our study with equal cooperation rates of around 65 and 66% for the two chambers. Cooperation rates range between 56 and 77% for the larger parties in the Swiss parliament and are higher for French speaking (79%) than for German speaking MPs (61%). The results reported in the regression analyses are based on a slightly smaller group of politicians and estimations for three reasons. First, and as mentioned, we only have reliable information about the policy preferences of the party electorate for the larger parties. Therefore, we had to exclude all estimations (54) made by six MPs from smaller parties. Second, we were obliged to exclude all estimations (27) of three further MPs who were reluctant to participate due to the length of the survey. They were given a shorter version, which did not require MPs to evaluate the electorate opinion. Finally, we had to exclude six further estimations as MPs sometimes did not evaluate the electorate opinion on each of the nine statements. In total, our analysis thus covers 142 MPs who made a total of 1278 estimations.^3^

Our measure of perceptual accuracy (PA) per policy statement per MP is constructed as follows:


PA=100−|agree_elect−agree_est|

where *agree_elect* is the percentage of respondents in the MPs’ party electorate rather or absolutely agreeing with the policy statement and *agree_est* the percentage of the party electorate which the MP estimates to agree with the statement.

The majority of studies in the field have used this approach to operationalize perceptual accuracy (e.g. Belchior, 2014; Broockman and Skovron, 2018). The mean perceptual accuracy is 79.82 (SD = 16.55) with remarkable differences across political parties and individual MPs.

### Independent variable

We use two different measures to capture whether MPs rely on policy information provided by interest groups. The first relies on data drawn from the official Swiss registry of MPs’ ties to interest groups, whereas the second relies on MPs’ self-reported data from a survey. Estimating separate models with these two measures allows us to address potential under-reporting that self-reported measures are prone to.

For both measures, we classified interest groups according to the Interarena coding scheme (Binderkrantz et al., 2015). It distinguishes between trade unions, business interest associations, occupational groups, identity, hobby/leisure, religious, and finally public interest groups. Most groups mentioned by MPs could be linked to an extensive dataset on interest groups participating in Swiss decision-making processes (Eichenberger, 2020; Gava et al., 2017). We aggregated business interest associations and occupational groups to a single business group category, and the remaining categories into a single citizen group category.

Our first and most detailed measure relies on the official registry of MPs’ ties to interest groups, published annually by the parliamentary services. It allows capturing the possibility that MPs rely on information from different types of interest groups depending on the statement. In Switzerland, MPs have to declare whether they engage in ‘permanent management or consultancy activities on behalf of Swiss or foreign interest groups’ (Art. 11d, Parliament Act). The 142 MPs comprised within the regression analyses held a total 734 ties to 596 unique interest groups at the time of data collection.^4^ When MPs hold ties to groups that are active in the same policy domain as the evaluated statement, then we consider these ties to capture MPs’ inclination to rely on information provided by (different types of) interest groups on this particular statement. To establish a match between the policy statements and the interest groups holding ties, we relied on groups’ participation in governmental consultation procedures. We coded the policy statements according to the policy topics suggested by the Comparative Agendas Project (CAP, see Table A1 in the Online Appendix). If MPs hold ties to groups that participated in governmental consultation procedures with the same CAP topic code as the policy statement, then we consider ties to capture MPs’ tendency to rely on information furnished by interest groups.

Our second measure relies on the aforementioned MPs survey, in which we asked MPs to indicate which interest group was the most useful for them in their political work. If MPs indicated, for instance, a citizen group as being most useful in their political work, then we consider them inclined to rely on information provided by citizen groups. Compared to the first measure, the categories are mutually exclusive: MPs can display a tendency to rely on information by no groups at all, business groups or citizen groups, but not both business and citizen groups at the same time. And compared to the first measure, MPs’ inclination to work with a specific type of group applies invariably across all statements.

Importantly, in both cases these ties capture a tendency or predisposition of MPs to rely on information provided by interest groups. The existence of a relevant tie alone does not necessarily imply an exchange of information. Rather, it captures MPs’ inclination to rely on information furnished by interest groups. As such, we see ties as a mere precondition for an exchange of information. The exchange itself only takes place if MPs make an effort to inform themselves about their electorate’s opinion. When they do so, and also display relevant ties, then this effort makes them draw on, among others, information provided by (different types of) interest groups. We consider the importance that MPs attach to a given statement as a proxy for the general effort undertaken to learn about electorate preferences. For each policy statement, we directly asked the MPs to assess its importance on a scale reaching from 0 (not at all important) to 10 (very important).

With regard to our first hypothesis, this means that we do not expect a main effect for citizen ties (as no information flows), but we expect a positive interaction term between citizen ties and importance. The position of the citizen group, which MPs learn about as they exchange information, represents a relatively unbiased estimator of the electorate’s opinion and thus strengthens the effect of importance (qua information) on perceptual accuracy. Inversely, and with regard to H2, we expect business ties to weaken the effect of importance on perceptual accuracy as MPs tend to identify the position of a subsection of the electorate with the position of their entire electorate. Again, we do not expect a main effect for business ties.

Moreover, it is important to point out that we consider MPs to report ties to ‘friendly’ groups in both the registry and the survey. For instance, ties to citizen groups working on migration issues (and thus relevant for statements 6, 15 and 16, see Table A1 in the Online Appendix) comprise ties to both pro- and anti-migration groups. We assume left-wing MPs to publicly announce ties to rather pro-migration groups, and right-wing MPs to entertain ties to rather anti-migration groups, lest they want to disgruntle their electorate. Therefore, and in line with H1, we expect ties to citizen groups to increase the perceptual accuracy of both left- and right-wing MPs.

As Table 1 illustrates, and with regard to the registry measure, 18% of MPs’ estimations are potentially informed by citizen groups. This share is considerably higher for estimations made by MPs from Left (28%) than Right (12%) parties. In line with previous studies, MPs from Right parties (26%) rely more heavily on business groups than MPs from Left parties (8%), with the sample share situated at 19% (Belchior, 2014).^5^ These figures are similar if we focus on the survey measure, with 26% (21%) of all estimations potentially informed by business (citizen) groups, though with starker differences between left- and right-wing MPs.

**Table 1.** Descriptive statistics.

	All MPs			Left-wing MPs			Right-wing MPs			
	N	Mean	St. Dev	N	Mean	St. Dev	N	Mean	St. Dev	Type
**Dependent variable**										
Perceptual accuracy	1272	79.91	16.45	449	83.95	14.85	823	77.71	16.86	Continuous
**MP-statement level variables**										
Registry: ties to citizen groups (H1)	1272	0.18		449	0.28		823	0.12		Dummy
Registry: ties to business groups (H2)	1272	0.19		449	0.08		823	0.26		Dummy
Survey										Categorical
Citizen groups (H1)	330	0.26		278	0.62		52	0.06		
Business groups (H2)	270	0.21		27	0.06		243	0.3		
No group	672	0.53		144	0.32		528	0.64		
Importance of statement	1272	5.39	3.21	449	5.96	3.34	823	5.08	3.1	Continuous
Self-reported specialization	1272	0.33		449	0.38		823	0.29		Dummy
Committee specialization	1272	0.14		449	0.14		823	0.15		Dummy
Undecided	1272	6.19	2.35	449	6.28	2.39	823	6.15	2.33	Continuous
Density	1272	329.11	207.45	449	330.38	209.54	823	328.42	206.43	Continuous
**MP level variables**										
Right-wing party (ref: left-wing)	142	0.65		50	0		92	1		Dummy
Delegate (ref: trustee)	142	8.23	1.88	50	8.68	1.38	92	7.99	2.07	Continuous
Female (ref: male)	142	0.26		50	0.5		92	0.13		Dummy
Experience	142	14.82	8.01	50	16.14	9.99	92	14.11	6.65	Continuous

Across the different statements, and when focusing on the registry measure, the average share of estimations potentially informed by interest groups is situated at 18.5%. There is, however, considerable variation in the share of these ‘informed’ estimations across the different statements. Certain policy statements, which MPs generally consider important and which concern policy domains in which MPs generally hold many ties, display a higher share of ‘informed’ estimations (see Figure A1 in the Online Appendix).

### Control variables

We introduce four (sets of) control variables (see also Table 1). First, MPs who consider themselves delegates must generally (feel obliged to) invest more resources into discerning their electorate’s preferences. We therefore asked MPs whether they see themselves more as trustees of the public (doing what they themselves think is best while keeping in mind the interests of the public) or as delegates (doing what the people want) on a scale from 0 (delegate) to 10 (trustee).

The second set of controls concerns MPs’ policy specialization as this might lead them to invest more efforts on an issue (Peeters et al., 2019). Specialists seek out technical information, some of which can be provided by interest groups, particularly business interest groups (e.g. trade journals). We use MPs’ committee membership as well as their self-reported specialization as controls to capture two dimensions of specialization: the institutional level and the (extra-parliamentary) background MPs might have on an issue. First, membership in legislative committees gives MPs the opportunity to specialize in a given issue area, which also makes them the targets of interest groups (Chaqués-Bonafont and Márquez, 2016; Marshall, 2015). We attributed each statement to the legislative committee likely responsible for it (see Table A1 in the Online Appendix). MPs seated in a committee relevant for a given statement are considered to be specialists. Secondly, we asked MPs to indicate the policy areas in which they consider themselves to be specialized. If the policy area matches with one of our statements, we code this as self-reported specialization (see Table A1 in the Online Appendix).

Thirdly, we control for MPs’ partisan affiliation. Research shows that left-wing MPs are generally better at estimating public opinion than right-wing MPs (Belchior, 2014; Broockman and Skovron, 2018). We broadly differentiate between MPs from left-wing parties (Socialist – SP, Greens, and Green-liberals – GLP) and those from right ones (Swiss People Party – SVP, Conservatives – BDP, Liberal-Radicals – FDP, and Christian Democrats – CVP). On the MP level, we further control for MPs’ experience in parliament (i.e. seniority) and their gender.

Finally, and on the statement level, we control for the overall salience of an issue in the public debate by including the share of undecided voters on a given policy statement from the population survey mentioned before. We expect higher shares of undecided voters to be associated with lower perceptual accuracy due to increased uncertainty. Moreover, we control for the density of the interest group community active on an issue. We expect a higher density to increase contact opportunities between MPs and interest groups and thus to increase perceptual accuracy. Density was measured on the basis of the number of groups active in consultation procedures in the policy domain of a given statement (Christe et al., 2016).

## Findings and discussion

### Bivariate analysis

**Figure 1.**
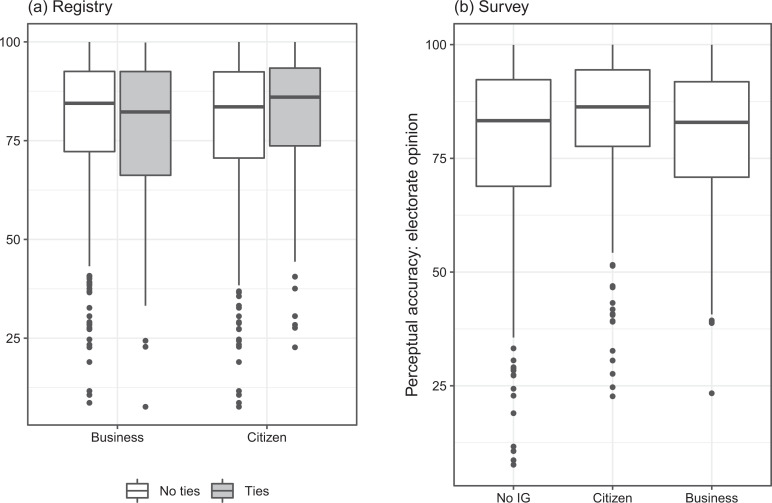
MPs’ perceptual accuracy, according to ties to interest groups: (a) registry and (b) survey.

Before delving into the multivariate analysis, we present some bivariate, descriptive statistics of our sample using both measures of interest group ties. In Figure 1, we can observe the accuracy of MPs’ estimations, depending on whether these estimations are (potentially) informed by different types of interest groups.

The left-hand panel (Figure 1(a)) focuses on MPs’ registry ties. As a reminder, MPs can hold board positions within both business and citizen groups at the same time, the categories are thus not mutually exclusive. Estimations informed by business groups (median = 82.3, mean = 78) are less accurate than those not informed by business groups (i.e. by citizen groups or no groups, median = 84.5, mean = 80.3). In contrast, estimations informed by citizen groups (median = 86, mean = 81.1) are more accurate than those informed by business groups or no groups (median = 83.6, mean = 79.5).

The right-hand panel (Figure 1(b)) focuses on MPs’ survey ties and reveals a similar picture. Estimations informed by citizen groups (median = 86.3, mean = 82.7) indeed appear more accurate than those not informed by interest groups (median = 83.3, mean = 78.7). The median value for estimations informed by business groups is situated at 82.8, below the respective values for estimations informed by citizen groups or not informed by interest groups. The mean value (79.8) slightly exceeds the mean value for estimations not informed by interest groups (78.5).

These descriptive statistics provide some prima facie evidence confirming both our hypotheses. However, bearing in mind that estimations informed by citizen groups are strongly associated to left-wing MPs (see Table 1), which have been shown to perceive their voters’ preferences more accurately (Belchior, 2014), a multivariate analysis is indispensable.

### Multivariate analysis

Table 2 shows the results of two multilevel regression models explaining MPs’ perceptual accuracy.^6^ Model 1 relies on the registry of ties to capture whether MPs’ estimations were informed by interest groups, whereas Model 2 relies on the MP survey. We discuss the results of these two measures in turn. In both models, the observations are nested within both MPs and policy statements and include those random effects.

**Table 2.** Multilevel regression models of MPs’ perceptual accuracy.

	Model 1	Model 2
(Intercept)	75.48*** (3.88)	77.51*** (4.31)
Level 1 (MP-statement)		
Registry citizen	−2.16 (2.37)	
Registry business	3.87* (2.26)	
Importance*Registry citizen	0.51 (0.36)	
Importance*Registry business	−0.75** (0.37)	
Survey citizen (ref: no group)		−6.34** (2.60)
Survey business (ref: no group)		1.46 (2.38)
Importance*Survey citizen		0.96*** (0.34)
Importance*Survey business		0.06 (0.37)
Importance	0.82*** (0.17)	0.51** (0.20)
Undecided	0.32 (0.31)	0.32 (0.35)
Density	0.00 (0.00)	0.00 (0.00)
Level 2 (MP)		
Right-wing party (ref: left-wing)	−5.93*** (1.34)	−6.90*** (1.65)
Self-reported specialization	−3.10*** (1.00)	−3.01*** (1.00)
Committee specialization	0.99 (1.31)	1.30 (1.31)
Delegate	0.45 (0.30)	0.46 (0.31)
Female (ref: male)	−1.18 (1.41)	−0.87 (1.44)
Experience	−0.10 (0.07)	−0.11 (0.07)
AIC	10655.59	10651.80
BIC	10743.11	10739.32
Log Likelihood	−5310.79	−5308.90
Num. obs.	1272	1272
Num. groups: MP	142	142
Num. groups: Statement	18	18
Var: MP (Intercept)	17.99	20.50
Var: Statement (Intercept)	5.33	7.28
Var: Residual	230.44	230.16

***p < 0.01; **p < 0.05; *p < 0.1.

Model 1, which focuses on the registry ties, displays a positive and statistically significant main effect for importance (qua information). Additional information thus increases the perceptual accuracy of MPs who draw this information from neither business nor citizen groups. We further observe a negative, strong interaction effect for importance and registry business ties. The positive effect of additional importance on MPs’ perceptual accuracy thus practically disappears if this additional information is (at least partially) drawn from business groups. Furthermore, the positive effect of additional importance on MPs’ perceptual accuracy appears to be strengthened if MPs draw their additional information (at least partially) from citizen groups, as indicated by the positive interaction term for importance and citizen ties. However, this effect is not statistically significant.

**Figure 2.**
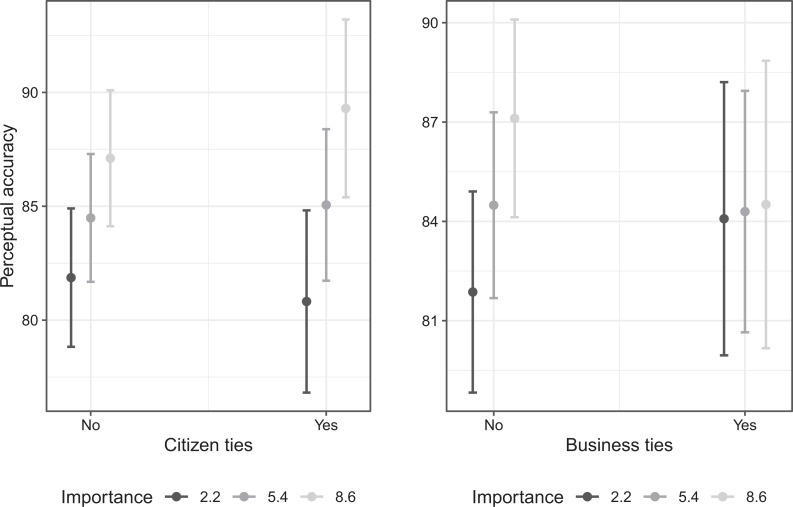
Effect of statement importance on MPs’ perceptual accuracy, depending on whether information (registry measure) is drawn from interest groups.

To facilitate the interpretation of these coefficients, Figure 2 displays MPs’ predicted perceptual accuracy at different levels of importance (mean ± 1 standard deviation), depending on whether MPs seek out information provided by citizen groups (Panel A) or business groups (Panel B). The left-hand panel shows that the effect of additional information appears strengthened if this additional information also stems from citizen groups. However, as this effect is not statistically significant we cannot confirm our first hypothesis on the basis of the registry data. The right-hand panel shows that more information does not increase MPs’ perceptual accuracy if this information is (partially) drawn from business groups. This confirms our second hypothesis.

Table 2 further reveals a statistically significant main effect for business ties. While this does not contradict our second hypothesis, we did not expect (neither business nor citizen) ties to exert an effect on perceptual accuracy when MPs make no effort to inform themselves. This could mean that MPs are affected by information from business groups even when they attach only very little importance to a statement.

When focusing on standardized coefficients (see Table A4, Model 1, in the Online Appendix), then it can be seen that the effect sizes for importance and its interaction term with business ties are comparable to the significant controls. Furthermore, if we relax the condition that ties must be relevant for a given statement (i.e. if we consider a tie to be relevant for all statements), then we observe substantially the same results (see Table A5 in the Online Appendix).

**Figure 3.**
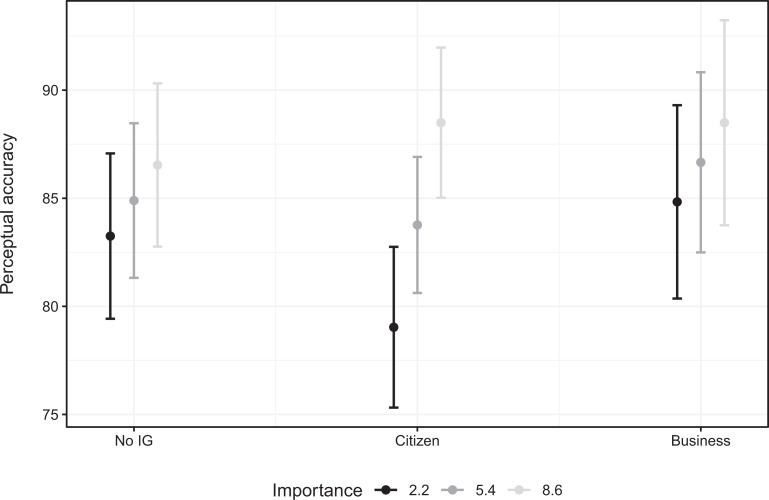
Effect of statement importance on MPs’ perceptual accuracy, depending on whether information (survey measure) is drawn from interest groups.

Model 2 (Table 2), which focuses on survey ties, provides somewhat contrasting findings. While the main effect of importance remains positive and significant, the interaction term between importance and mentioning a business group is now (very narrowly) positive (rather than negative as in Model 1) and not statistically significant. However, the interaction effect between importance and information stemming from citizen groups is now not only positive (as in Model 1), but also statistically significant. That is, when MPs rely on information provided by citizen groups in their effort to inform themselves about their electorate’s preferences, then the effect of additional information on MPs’ perceptual accuracy is considerably strengthened (see Figure 3). Model 2 thus confirms our first hypothesis, but not our second.

Moreover, we observe a negative, statistically significant main effect for information provided by citizen groups. That is, when MPs attach no importance to a statement (and when would expect them *not* to receive or seek out information from interest groups in the first place), then ties to citizen groups appear to decrease MPs’ perceptual accuracy.

Again, when focusing on standardized coefficients (see Table A4, Model 2, in the Online Appendix), it can be seen that the effect sizes for importance and its interaction term with citizen ties are comparable to the significant controls (see below).

Before attempting to (partially) resolve some of the apparent inconsistencies between our two models, we first discuss the findings with regard to relevant control variables.

First, MPs’ partisan affiliation exerts a strong, statistically significant effect across both models. Estimations made by right-wing MPs tend to be considerably less accurate than those made by their left-wing counterparts. This resonates with findings made in the US (Broockman and Skovron, 2018) and Portuguese contexts (Belchior, 2014). It has been argued that these differences also reflect the ties that these respective politicians have with specific types of groups. However, our results show that left-wing MPs display a higher perceptual accuracy even when controlling for ties to citizen groups. Inversely, right-wing MPs display a lower perceptual accuracy even when controlling for ties to business groups. Partisan affiliation thus does not simply capture ties to business and citizen groups, something proper to MPs’ party membership appears to be influencing their perceptual accuracy.

Secondly, specializing in a specific policy domain is likely to be correlated to holding ties to interest groups, business interest groups in particular. Our results show self-reported specialization exerts a negative, statistically significant effect on MPs’ perceptual accuracy. MPs perhaps tend to project their opinion onto their voters when considering themselves specialists in a given policy domain (Clausen et al., 1983; Fisher and Keil, 2016; Holmberg, 1999). Importantly, they do so even when controlling for ties to interest groups.

### Social desirability bias

We now return to Table 2 and the absence of a negative interaction between importance and business ties in Model 2. This finding might be related to a certain reluctance on behalf of MPs to acknowledge working with interest groups in general, and with business groups, which (on average) defend narrower interests than citizen groups, in particular. It could hence be the case that many MPs report *not* to draw on information stemming from business groups in our survey data when in fact they do.

Because we have detailed data from the official registry, we can directly test this explanation for our diverging findings. We compared MPs’ ties as declared in the registry to the groups they announced in the survey. As concerns the registry ties, we considered only ties to politically active groups, that is, groups that participated in governmental consultation procedures.^7^ MPs with more ties to business than citizen groups belong to the ‘Business’ category. We would expect such MPs to announce a business group as the most useful interest group they collaborate with. Inversely, MPs with more ties to citizen than to business groups belong to the ‘Citizen’ category.^8^ If MPs hold no ties, they belong to the ‘No IG’ category.

Table 3 shows that roughly 42% of all MPs predominantly tied to citizen groups announced no group in the politicians’ survey. That is, 42% of all MPs tied to at least one politically active citizen group declared *not* to be working with an interest group at all. While this shows that even MPs with ties to citizen groups refrain from announcing their ties in surveys, the share of MPs predominantly tied to business groups and declaring *not* to be working with interest groups at all is considerably higher, situated at 65%. A chi-square test reveals a statistically significant association between group type as declared in the registry and group type as declared in the survey: *X*^2^ (4, *N* = 149) = 19.0, *p* < .001. However, a glance at the standardized residuals (Table 3, in parentheses) reveals that this association is mainly driven by MPs tied to citizen groups (according to the registry). In fact, they announced no groups (in the survey) less frequently than expected and announced citizen groups more frequently than expected (under the null-hypothesis that there is no association between group type as declared in the registry and group type as announced in the survey). MPs tied to business groups, on the other hand, announced no groups (in the survey) more frequently than expected and in fact did not announce business groups more frequently than expected.

**Table 3.** MPs’ registry ties compared groups declared in survey (row percentages).

		Survey			
		No IG	Citizen	Business	N
Ties	Business	64.6% (1.84)	10.4% (−3.12)	25% (1.18)	48 (100%)
	Citizen	41.9% (−2.43)	45.2% (4.26)	12.9% (−1.71)	62 (100%)
	No IG	59% (0.77)	17.9% (−1.46)	23.1% (0.66)	39 (100%)

Standardized residuals in parentheses.

Two MPs excluded from the analysis as they held an equal number of relevant ties to interest groups which displayed the same average number of participations in consultation procedures.

These results suggest that MPs with ties to business groups tend to underreport this collaboration in surveys, at least to a stronger extent than MPs tied to citizen groups. This strongly echoes recent findings, according to which social desirability bias can seriously affect the validity of legislative surveys (Bundi et al., 2018). Furthermore, when declaring to work with business groups, MPs mainly announced peak-level organizations. In Switzerland, with its neo-corporatist heritage, these peak-level organizations represent important and publicly recognized actors. Perhaps this further conveys MPs’ reluctance to announce ties with the more sectional, branch-level organizations. Among the MPs announcing no ties to business groups, we might find many MPs in fact have ties to branch-level organizations.

A certain reluctance on behalf of MPs to announce their ties to sectional business groups in the survey might hence explain why additional information drawn from business groups does not appear to suppress the positive impact of additional information drawn from alternative sources (newspapers, time spent with constituency, peers, etc.) in Model 2.

## Conclusion

MPs’ ties to interest groups can raise the problem of divided loyalties, of MPs choosing to vote against their electorate. Recent research has shown that this problem mostly concerns MPs tied to business groups, whereas ties to citizen groups actually increase MPs’ propensity to vote in their electorate’s interest (Giger and Klüver, 2016; Gilens and Page, 2014; Klüver and Pickup, 2019). We investigated the causal mechanism behind these findings, by studying interest groups’ influence on MPs’ perceptions of their party electorate’s opinion.

By differentiating between business and citizen groups, we focused on the important distinction between narrowly and broadly shared interests, firmly established within the literature on interest groups (Baroni et al., 2014). Our results underline that the distinction between citizen and business groups is crucial to understanding how interest groups shape MPs’ perceptual accuracy. When relying on our survey to measure ties between interest groups and MPs, then citizen groups appear to strengthen MPs’ perceptual accuracy, even when controlling for partisan affiliation. Policy expertise drawn from citizen groups, which serve broadly shared interests, allows MPs to learn about the opinion of a large part of their electorate. When relying on the official registry to measure ties, this positive effect is no longer statistically significant. However, the registry ties, unlike the survey ties, suggest that business groups, which defend narrowly shared interests, decrease MPs’ perceptual accuracy, even when controlling for MPs’ partisan affiliation. This means that MPs could be voting against their electorate ‘in good faith’, confounding interest groups’ and constituents’ interests rather than privileging one over the other.

The inconsistencies between our models appear, at least partially, related to a problem of misreporting in legislative surveys. MPs with officially validated ties to interest groups in general, and business groups in particular, in fact underreported these ties in our survey. This has made it difficult to probe the robustness of our findings regarding the effect of business ties. Incidentally, however, this misreporting bias, which is the likely consequence of MPs providing socially desirable rather than accurate answers, attests to the importance of relying on ‘objective’ rather than self-reported data when studying interest groups’ access and influence (see, for instance, the efforts undertaken by Bernhagen et al., 2014). If MPs hesitate to report their ties to business groups, then business groups might also hesitate to report their ties to MPs.

As concerns the study of MPs’ perceptual accuracy, this social desirability bias is not easily tackled. Unless we are able to identify and contact all interest groups that potentially informed MPs’ estimations, we are obliged to rely on MPs’ declarations. And if interest groups are prone to misreport as well, the only way out of this dead end is validated data through a lobbying registry. This brings us to three shortcomings which future research might wish to address. The first two address our measures of the independent and dependent variables respectively, while the third point concerns extensions to our research design.

First, further research could expend more resources on devising a more direct measure to capture whether MPs’ receive information from interest groups when dealing with policy issues. In our case, we could have asked MPs to list the relevant groups for each policy statement. However, this would have considerably increased the cognitive burden of an already lengthy survey. Another promising route, which might also allow addressing misreporting bias, leads towards a more experimental design, in which MPs are ‘treated’ to vignettes displaying information by different types of interest groups. Perhaps this allows tackling any misreporting bias.

Secondly, we did not measure *directional* perceptual accuracy. We argue that reliance on information from business groups should ‘pull’ MPs’ estimations of their electorate’s opinion *towards* the interest group’s position. For instance, if MPs rely on information from pro free trade business groups, then they should perceive their electorate as being more (rather than less) pro free trade than it is in actuality. Our measure does not take this into account. Strictly speaking then, our results could mean that reliance on information from pro free trade business groups actually leads MPs to perceive their electorate as *less* pro free trade (more protectionist) than it actually is. Taking into account interest groups’ positions would certainly allow us to build a more precise measure, but we do not see why MPs with business ties should consider their electorate as less business friendly than it actually is. Nevertheless, a measure of directional perceptual accuracy would allow to dispel doubts. Furthermore, on a conceptual level, we focused on whether MPs can accurately perceive their electorate’s opinion on a given statement. Further research could also focus on how interest groups affect MPs’ perceptual accuracy of different issues’ saliency among the party electorate. This might allow testing one of the core assumptions behind the theory of ‘quiet politics’ (Culpepper, 2011), namely whether MPs correctly perceive the saliency of an issue among the party electorate.

Finally, our research design would benefit from an inclusion of both more countries and issues. A cross-country design allows a better assessment of institutional variables’ impact on the link between interest groups and MPs’ perceptual accuracy. Swiss MPs can make use of powerful agenda-setting and law-making instruments, but they receive relatively little pay as their legislative mandate is part-time and incidental to a principal professional activity. MPs are probably more dependent on information drawn from interest groups than MPs in highly professionalized parliaments, and thus more likely to confound interest groups’ and voters’ preferences.

An inclusion of further issues and thus statements would allow us to test a broader set of issue-level characteristics, including, for instance, issue dimensionality. At the same time, it would enable a more stringent test of the issue-level controls already included in our models.

## Supplemental material

Supplemental Material, sj-pdf-1-ppq-10.1177_1354068821997079 - Do interest groups bias MPs’ perception of party voters’ preferences? Click here for additional data file. Supplemental Material, sj-pdf-1-ppq-10.1177_1354068821997079 for Do interest groups bias MPs’ perception of party voters’ preferences? by Steven Eichenberger, Frédéric Varone and Luzia Helfer in Party Politics

## Notes

1. This hinges upon conservative groups’ positions diverging considerably from those of the majority of an MP’s electorate, which is less likely to be the case in rather conservative electoral districts.

2. Data available at: https://doi.org/10.26037/yareta:4qoe7wpc45amtk2duqwgjxzu2m.

3. (142 MPs * 9 statements) − 6 estimations for which MPs did not evaluate the electorate’s opinion.

4. We took ties declared at the beginning of the year of data collection (2018) as reference. For the eight MPs who joined parliament during the year, we used the ties declared at the beginning of 2019 (as the registry of ties is updated only at the beginning of the calendar year). We excluded board positions held within companies (private and public), family foundations and museums from the present analysis.

5. When bearing in mind that most MPs entertain regular contact with interest groups (see, for instance, Wonka, 2017), these figures appear low. However, we focus on contacts pertaining to a limited number of statements rather than contacts in general. In fact, 40% (46%) of all MPs display a tendency to rely on business (citizen) group information with regard to *at least one* of the assessed policy statements. Had we asked MPs to assess more policy statements, then this figure would likely be even higher.

6. Regression tables were produced with the texreg R package (Leifeld, 2013).

7. We connected the data on MPs’ ties to data on interest groups’ participation in consultation procedures (Christe et al., 2016).

8. When MPs held an equal number of business and citizen ties, we attributed the MP to the group category with the higher average number of participations in consultation procedures.
