# Supplementary material for: Do interest groups bias MPs’ perception of party voters’ preferences?
Source: Party Politics. 2021 Apr 15;28(3):567–79. doi: 10.1177/1354068821997079 (PMC9036159; doi:10.1177/1354068821997079)
Supplement: Supplemental Material, sj-pdf-1-ppq-10.1177_1354068821997079 - Do interest groups bias MPs’ perception of party voters’ preferences? [file sj-pdf-1-ppq-10.1177_1354068821997079.pdf]

Appendix

Tables

For Peer Review

Table A1. Policy statements assessed by MPs, with associated CAP topics, legislative committees and self-reported specialization categories

ID	Batch	Statement	CAP topic(s)	Committee	Self-reported specialization
1	A	Switzerland needs to buy new fighter jets.	Defence	SPC	Defense & military
2	A	Jobs in my Canton need to be reserved for people residing my Canton.	Labour and Employment; Economic and Commercial Regulation	EATC	Labour
3	A	The concerned Cantons need to allow the hunt of wolves that attack flock.	Environment	ESPEC	Environment, energy, transportation, spatial planning
4	A	Hospitals need to have a \Babyklappe\ where parents can leave their infant anonymously.	Social Affairs	SSHC	Ethical questions
5	A	Sexual harassment at work needs to be punished more severely.	Rights, Liberties and Minority Issues	LAC	Justice
6	A	Switzerland should only accept well-educated immigrants.	Immigration and Integration	PIC	Immigration & asylum
7	A	Citizens should be able to participate in federal elections via internet.	Government and Public Administration	PIC	Civil rights & political institutions
8	A	Taxes on high-income should be raised while taxes on low-income should be reduced.	Macroeconomics; Economic and Commercial Regulation	EATC	Public administration & taxes
9	A	The pension age needs to be raised to 67.	Labour and Employment; Economic and Commercial Regulation	SSHC	Social affairs
10	B	Unused civil protection shelters should be definitely closed.	Defence	SPC	Defense & military
11	B	Elderly employees need to be better protected from dismissal.	Labour and Employment; Economic and Commercial Regulation	EATC	Labour
12	B	Private households should be able to freely choose their electricity provider.	Energy; Environment	ESPEC	Environment, energy, transportation, spatial planning
13	B	Same-sex couples who have registered their partnership should be allowed to adopt children.	Rights, Liberties and Minority Issues	LAC	Ethical questions
14	B	The police needs to prevent unauthorized demonstrations at all costs.	Justice and Legal Affairs	SPC	Justice
15	B	My Canton should spend more for the integration of asylum seekers.	Immigration and Integration	PIC	Immigration & asylum
16	B	Foreigner residing in Switzerland for more than 10 years should be given the right to vote at the cantonal level.	Immigration and Integration	PIC	Civil rights & political institutions
17	B	Wedded people need to be assessed separtaely for taxation.	Social Affairs	EATC	Public administration & taxes
18	B	My canton should create a cantonal health insurance institution for ist residents.	Health	SSHC	Social affairs

[a] Each MP evaluated only one batch of statements (either A or B). 609 estimations thus pertain to statements from batch A and 663 to statements from batch B (which makes for a total 1272 observations, see regression tables).

[b] The CAP topics assigned to each statement were used to establish whether a tie to an interest group (also coded according to the CAP topics) is relevant for a specific statement. The committees and self-reported specialization categories assigned to each statement allow establishing MPs' committee and self-reported specialization.

[c] EATC: Economic affairs and taxation; ESPEC: Environment, spatial planning and energy; LAC: Legal affairs; PIC: Political institutions; SPC: Security policy; SSHC: Social security and health

For Peer Review

Table A2. Number of voters' opinions per party electorate.

Party	min.	max.
SP	352	425
SVP	357	407
FDP	301	343
CVP	146	174
Greens	101	131
GLP	82	123
BDP	51	76
**Mean (SD)**	**307 (114)**	

For Peer Review

Table A3. Multi-level regression models of MPs' perceptual accuracy (no interactions).

	Model 1	Model 2
(Intercept)	76.00***	76.61***
	(3.85)	(4.22)
Level 1 (MP-statement)		
Registry citizen	0.64	
	(1.31)	
Registry business	0.06	
	(1.31)	
Survey citizen (ref: no group)		−0.80
		(1.72)
Survey business (ref: no group)		1.88
		(1.48)
Importance	0.77***	0.79***
	(0.15)	(0.15)
Undecided	0.30	0.30
	(0.31)	(0.34)
Density	0.00	0.00
	(0.00)	(0.00)
Level 2 (MP)		
Right-wing party (ref: left-wing)	−6.01***	−6.95***
	(1.34)	(1.64)
Self-reported specialization	−3.06***	−2.99***
	(1.00)	(1.00)
Committee specialization	1.16	1.20
	(1.31)	(1.32)
Delegate	0.44	0.42
	(0.30)	(0.31)
Female (ref: male)	−0.98	−0.83
	(1.41)	(1.43)
Experience	−0.10	−0.11
	(0.07)	(0.07)
AIC	10657.48	10655.80
BIC	10734.71	10733.03
Log Likelihood	−5313.74	−5312.90
Num. obs.	1272	1272
Num. groups: MP	142	142
Num. groups: Statement	18	18
Var: MP (Intercept)	18.19	19.75
Var: Statement (Intercept)	5.10	6.62
Var: Residual	231.54	232.05

***$p < 0.01$; **$p < 0.05$; *$p < 0.1$

For Peer Review

Table A4. Multi-level regression models of MPs' perceptual accuracy (standardized coefficients).

	Model 1	Model 2
(Intercept)	0.00	0.00
	(0.05)	(0.05)
Level 1 (MP-statement)		
Registry citizen	−0.05	
	(0.05)	
Registry business	0.09*	
	(0.05)	
Importance*Registry citizen	0.08	
	(0.05)	
Importance*Registry business	−0.11**	
	(0.05)	
Survey citizen (ref: no group)		−0.17**
		(0.07)
Survey business (ref: no group)		0.04
		(0.06)
Importance*Survey citizen		0.18***
		(0.06)
Importance*Survey business		0.01
		(0.05)
Importance	0.16***	0.10**
	(0.03)	(0.04)
Undecided	0.05	0.05
	(0.04)	(0.05)
Density	0.03	0.03
	(0.04)	(0.05)
Level 2 (MP)		
Right-wing party (ref: left-wing)	−0.17***	−0.20***
	(0.04)	(0.05)
Self-reported specialization	−0.09***	−0.09***
	(0.03)	(0.03)
Committee specialization	0.02	0.03
	(0.03)	(0.03)
Delegate	0.05	0.05
	(0.03)	(0.03)
Female (ref: male)	−0.03	−0.02
	(0.04)	(0.04)
Experience	−0.05	−0.05
	(0.03)	(0.04)
AIC	3515.65	3576.55
BIC	3603.18	3664.07
Log Likelihood	−1740.83	−1771.28
Num. obs.	1272	1272
Num. groups: MP	142	142
Num. groups: Statement	18	18
Var: MP (Intercept)	0.07	0.07
Var: Statement (Intercept)	0.02	0.03
Var: Residual	0.84	0.84

$^{***}p < 0.01$; $^{**}p < 0.05$; $^{*}p < 0.1$

For Peer Review

Table A5. Multi-level regression models of MPs' perceptual accuracy (no relevance condition).

	Model 1
(Intercept)	75.60***
	(4.49)
Level 1 (MP-statement)	
Registry citizen	−1.77
	(2.42)
Registry business	0.86**
	(0.40)
Importance*Registry citizen	0.27
	(0.37)
Importance*Registry business	−0.49*
	(0.29)
Importance	2.79
	(2.01)
Undecided	0.32
	(0.31)
Density	0.00
	(0.00)
Level 2 (MP)	
Right-wing party (ref: left-wing)	−6.14***
	(1.35)
Self-reported specialization	−3.04***
	(1.00)
Committee specialization	1.14
	(1.31)
Delegate	0.43
	(0.30)
Female (ref: male)	−1.14
	(1.42)
Experience	−0.10
	(0.07)
AIC	10658.08
BIC	10745.60
Log Likelihood	−5312.04
Num. obs.	1272
Num. groups: MP	142
Num. groups: Statement	18
Var: MP (Intercept)	18.39
Var: Statement (Intercept)	5.12
Var: Residual	230.76

***$p < 0.01$; **$p < 0.05$; *$p < 0.1$.

[a]If MPs announce ties to e.g. citizen groups in the registry, estimations might be informed by citizen groups, regardless of statement.

For Peer Review

Table A6. Multi-level regression models of MPs' perceptual accuracy (with only two controls).

	Model 1	Model 2
(Intercept)	79.82***	81.58***
	(1.59)	(1.99)
Level 1 (MP-statement)		
Registry citizen	−1.98	
	(2.38)	
Registry business	4.14*	
	(2.28)	
Survey citizen (ref: no group)		−5.51**
		(2.59)
Survey business (ref: no group)		2.12
		(2.39)
Importance	0.81***	0.53***
	(0.17)	(0.20)
Importance*Registry citizen	0.49	
	(0.36)	
Importance*Registry business	−0.74**	
	(0.37)	
Importance*Survey citizen		0.93***
		(0.34)
Importance*Survey business		0.03
		(0.37)
Level 2 (MP)		
Right-wing party (ref: left-wing)	−5.79***	−6.59***
	(1.26)	(1.56)
Self-reported specialization	−2.68***	−2.53***
	(0.96)	(0.96)
AIC	10725.12	10712.10
BIC	10781.81	10768.79
Log Likelihood	−5351.56	−5345.05
Num. obs.	1279	1279
Num. groups: MP	143	143
Num. groups: Statement	18	18
Var: MP (Intercept)	21.02	22.27
Var: Statement (Intercept)	6.55	7.20
Var: Residual	232.61	232.34

$^{***}p < 0.01$; $^{**}p < 0.05$; $^{*}p < 0.1$

For Peer Review

Figures

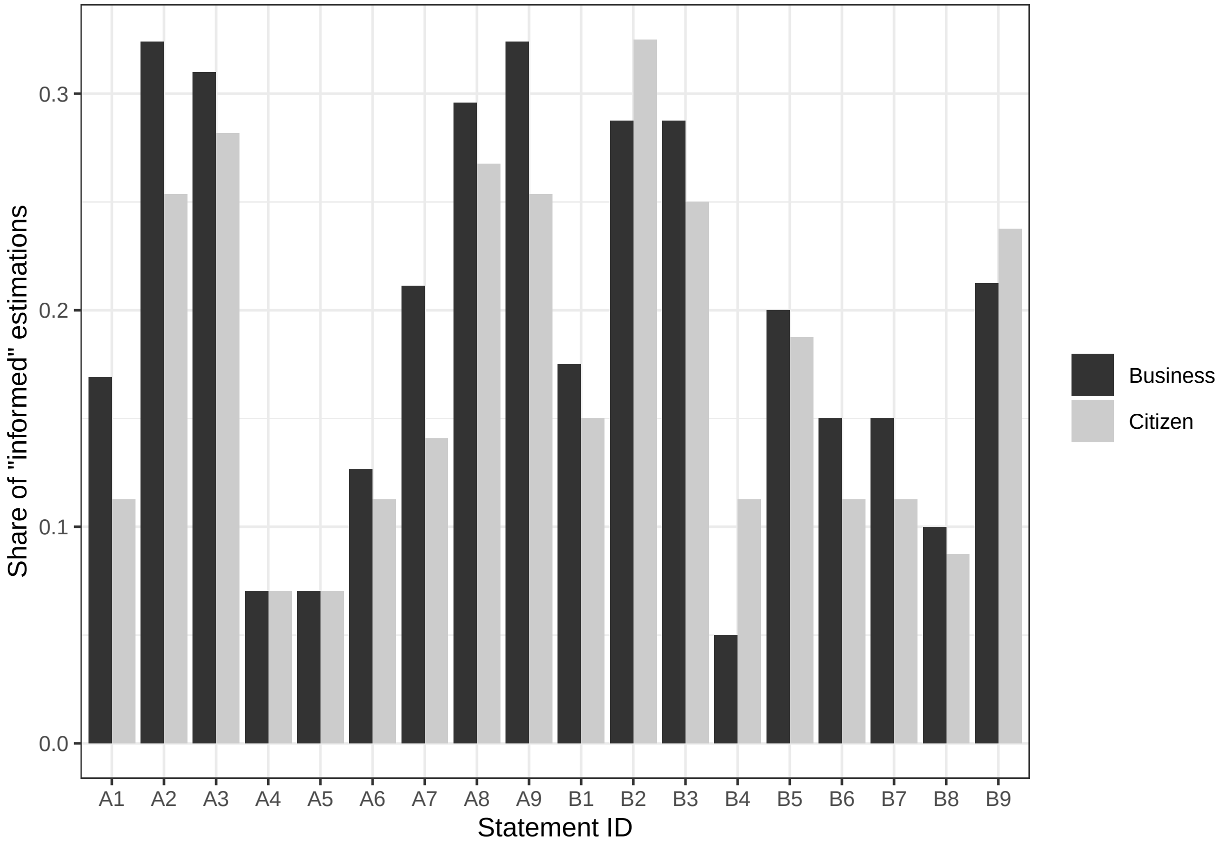

Figure A1. Share of MPs' estimations informed by interest groups.
